# State of the science on controversial topics: orthodontic therapy and gingival recession (a report of the Angle Society of Europe 2013 meeting)

**DOI:** 10.1186/2196-1042-14-16

**Published:** 2013-07-11

**Authors:** Ama Johal, Christos Katsaros, Stavros Kiliardis, Pedro Leito, Marco Rosa, Anton Sculean, Frank Weiland, Björn Zachrisson

**Affiliations:** Centre for Oral Growth and Development, 5th Floor, Institute of Dentistry, Barts and the London School of Medicine and Dentistry, Turner Street, Whitechapel, London, E1 2AD UK

**Keywords:** Gingival recession, Orthodontics treatment

## Abstract

**Background:**

Controversy exists in the literature between the role of orthodontic treatment and gingival recession. Whilst movement of teeth outside the alveolar bone has been reported as a risk factor for gingival recession, others have found no such association.

**Findings:**

The Angle Society of Europe devoted a study day to explore the evidence surrounding these controversies. The aim of the day was for a panel of experts to evaluate the current evidence base in relation to either the beneficial or detrimental effects of orthodontic treatment on the gingival tissue.

**Conclusions:**

There remains a relatively weak evidence base for the role of orthodontic treatment and gingival recession and thus a need to undertake a risk assessment and appropriate consent prior to the commencement of treatment. In further prospective, well designed trials are needed.

## Findings

### Introduction

Gingival or soft tissue recessions are defined as the displacement of the marginal tissue apical to the cemento-enamel junction and can affect the labial, lingual and/or interproximal areas [1]. Gingival recession is reported to increase in both severity and prevalence with age, with greater than 90% of adults aged 50 years and above demonstrating its presence [2]. The labial aspect of the mandibular incisors and maxillary molars is being most frequently affected [3]. The aetiology is incompletely understood and thought to be multifactorial in nature, with both predisposing and precipitating factors implicated. The former constitutes anatomic and morphological characteristics, such as alveolar bone dehiscence, thin buccal mucosa, crowding, presence of aberrant fraenula and ectopic tooth eruption. Precipitating factors lead to an acceleration of the defect, such as traumatic tooth brushing and piercing [4]. Controversy exists in the literature between the role of orthodontic treatment and gingival recession. Whilst, movement of teeth outside the alveolar bone has been reported as a risk factor for gingival recession [5], others have found no such association [6, 7].

Thus, the aim of the day was for a panel of experts to evaluate the current evidence base in relation to either the beneficial or detrimental effects of orthodontic treatment on the gingival tissue.

## Method

The format of the day was that the *morning session* consisted of a number of interrelated presentations, by leading clinicians from both within the Society and an invited guest speaker (Professor Anton Sculean), followed by a 5-min open discussion. Each presentation raised a number of questions for further discussion in the afternoon breakout sessions.

The *afternoon session* involved the ASE members forming into seven breakout groups and an appointed chair, with each group being assigned to address one or more questions arising from the morning presentations. Each breakout group chair presented back to the whole group their conclusions during the final session of the evening. As the findings in relation to each question were addressed, a very interactive whole group discussion followed and consensus viewpoints sought.

The day was summarised, and a number of conclusions reached.

### Aetiology, classification, epidemiology and need for treatment of gingival recession

The presentation focussed not only on the aetiology, classification, epidemiology and need for treatment but also highlighted the indications for treatment, which could include aesthetic concerns, hypersensitivity and the need to improve oral hygiene. The benefits of Miller's classification of gingival recession were presented in terms of permitting an evaluation of prognosis to be undertaken [8]. The importance of optimal plaque control and aesthetics with complete root coverage were highlighted as the treatment goals.

### Orthodontic treatment and gingival recessions: what is the evidence from animal experimental studies?

The presentation highlighted the limited scientific quality of research to date, with studies being confined to small numbers of either dogs or monkeys. The evidence in relation to the effect on the gingival tissues of moving teeth in the following manner was presented: outside the cortical plate and the observed changes with and without moving the teeth back. It appeared that moving the teeth outside the cortical plate and retaining them in this position resulted in loss of both bone and soft tissues. Whilst moving them back to within the alveolus was accompanied by a variable (up to 50%), gain in bone but no soft tissue benefit was observed.

### Orthodontic treatment and gingival recessions: what is the evidence from clinical studies?

The benefit of using light controlled orthodontic forces to promote favourable gingival tissue and bony changes in particular intrusion and extrusion were highlighted. The findings from the systematic review by Joss-Vassalli et al. [9] were presented, which demonstrated a weak evidence base (with studies being retrospective in nature and of low-to-moderate value of evidence), weak methodology, short-term follow up and a significant number of confounding variables, which were not controlled for. The authors reported contradictory results regarding a possible statistically significant correlation between incisor proclination and subsequently resulting gingival recession and recommended caution in interpreting the findings. Evidence linking incisor decompensation in Class III malocclusion and the importance of the mandibular symphyseal anatomy were highlighted, in conjunction with identifying potential risk factors.

### Aspects on the envelope of the lower anterior alveolar process: incisor proclination by orthodontic means or distraction osteogenesis?

The concept of an alveolar ‘envelope’ was proposed, within which the teeth should be maintained. The cortical bone of the alveolar process was considered to be the anatomic border of this envelope. Thus, the prospect of alveolar distraction in the anterior region was discussed as a means of ‘transporting’ the alveolar process with the teeth, creating better conditions to avoid recessions. This was evaluated in a comparative controlled longitudinal study of orthodontic treatment alone versus orthodontic treatment in conjunction to distraction [10]. Overall all groups demonstrated an increase in clinical crown height and a variable degree of gingival recession over the follow-up period, no matter if the proclination was achieved with pure orthodontics or by means of destruction. No definable value of incisor proclination leading to gingival recession could be identified. However, severe proclination appeared to be contributory. It appeared that respect for the bony envelope alone was not sufficient to avoid recessions. The importance of the soft tissue envelope could be considered in the future as a contributing factor.

### Gingival recession in orthodontics: a clinical matter

The importance of early correction of any observed detrimental effect on the gingival tissues or alveolar bone during orthodontic treatment, through a combination of appropriate root torque and optimal patient oral hygiene, was highlighted. In addition, the need to identify any risk factors, such as tissue biotype and the thickness of the alveolar bone was also emphasised to minimise gingival recession. The large individual variation of the soft tissue following inappropriate orthodontic movements, such as dento-alveolar expansion and derotation was highlighted, as well as the value of orthodontic intrusion and correction of the traumatic occlusion, in addition to the effectiveness/efficiency of interceptive treatment during the mixed dentition in guiding the correct eruption of the permanent teeth into the periodontal envelope.

The development of gingival recession during or after orthodontic treatment would be a significant clinical problem. This not only highlights the need to undertake a risk assessment before treatment is commenced, with appropriate consent, but also to be aware during treatment for signs of recession. In particular, a baseline line assessment of the gingival margin heights in addition to the patient's age, biotype, planned arch expansion and the width of the alveolus should be taken into account prior to commencing treatment. Gingival recession occurring after orthodontic treatment has the potential for causing aesthetic or psychological concerns and hypersensivity. These concerns relate directly to the age of the patient, as they are likely to be progressive in nature, and the severity of gingival recession in terms of the long-term prognosis of the tooth in question.

A number of predisposing and precipitating factors are considered important identifiable risk factors for gingival recession in relation to orthodontic treatment [11]. Predisposing factors include: anatomical and morphological characteristics, such as alveolar bone dehiscence, gingival biotype, skeletal pattern, narrow symphysis and ectopic tooth eruption or morphology. Precipitating factors lead to an acceleration of the defect, such as traumatic tooth brushing, traumatic overbite, age, smoking, parafunctional habits, pregnancy and piercing. In addition and perhaps equally important are inappropriate treatment mechanics, such as arch expansion, with excessive proclination and the use of RME in adult patients. Care should also be taken when decompensating a class III incisor relationship in preparation for surgery and aligning ectopic/transposed teeth. One could consider the acronym ABEF to help take into account the risk factors:

A: Anatomy of the alveolar bone and proximity of the root to the cortical plates
B: Biotype
E: Environment (oral hygiene, habits, poor brushing, poor orthodontic mechanics, active lingual retainers)
F: Functional matrix

It is equally important to recognise that a number of orthodontic procedures, such as the judicial use of dental extractions, interproximal enamel reduction, correct root torque, selective grinding and if indicated treatment in the mixed dentition can act to retain the roots within the alveolar bone and thereby reduce root prominence and the risk of gingival recession. They may also allow creeping attachment and, if planned, a better future surgical site. Thus, amongst the reported benefits of orthodontic treatment in relation to gingival recession are as follows:

1. Self-maintaining oral hygiene
2. Crown alignment within the dento-alveolar envelope
3. Removal of occlusal trauma
4. Root alignment within the bone
5. A hopeless tooth is not a useless tooth - the value of a periodontal opinion is important, as such teeth can be utilised to enhance bone and/or soft tissue anatomy before insertion of implants

To minimise the risk of gingival recession and maximise the benefit of the orthodontic treatment, the orthodontist must be aware of the risk factors identified above, and it is time that we, as professionals, take into account more than just the crown but perhaps more importantly the roots and their proximity to the cortical plates. Thus, the mechanics or treatment modalities that could be employed to minimise the risk of recession include the following:

1. Maintain good oral hygiene throughout orthodontic treatment and identify potential risk factors
2. Eliminate potential causes of recession (piercing, smoking, traumatic toothbrushing)
3. Avoid uncontrolled dento-alveolar expansion and maintain arch form
4. Customise bonding and mechanics
5. Modify tooth anatomy whenever indicated
6. Consider segment arch mechanics
7. Create space before using it and use it wisely
8. Consider atypical extractions, e.g. compromised teeth
9. Avoid jiggling because it may cause periodontal problems
10. Treat early *(interceptive procedures and treatment in mixed dentition)*
11. Re-educate the patient in their oral hygiene technique after the end of treatment.

### Development of gingival recessions in the post-orthodontic treatment period

The recent findings of a series of retrospective studies designed to evaluate the long-term effect of orthodontic treatment on labial gingival recession 5 years post-treatment were presented. Change in lower incisor inclination did not affect the development of labial gingival recession [12]. Retainer type was not an influencing factor in labial gingival recession, but age at the end of treatment was the greatest predictor [13]. When comparing a group of orthodontically treated patients to a matched untreated control group, the proportion of subjects with recessions was consistently higher in treated patients than in untreated subjects. In orthodontically treated patients, lower incisors seem to be the most *susceptible* to the development of labial gingival recession [14]. The possible development of gingival recession due to active multistranded lingual retainers was discussed in light of current findings [15, 16].

### Indications for increasing soft tissue thickness prior to orthodontic treatment

A treatment protocol for managing patients with gingival recession was presented, in which, ideally further incisor proclination should be avoided. However, in patients requiring pre-surgical decompensation, pre-prosthetic preparation or where a non-extraction approach is judged as necessary: the importance of ensuring optimal oral hygiene and using a free gingival graft prior to the planned orthodontic tooth movement should be considered. The preferred approach in these susceptible patients should be to again ensure optimal oral hygiene, align the roots within the alveolar envelope, avoiding proclination and to re-evaluate the need for a mucogingival graft after treatment.

### Treatment of gingival recession: state of the art

The presentation identified the following general factors, which influenced treatment outcome: tooth (crown morphology), soft tissues (biotype), bone morphology (presence or absence of interproximal bone), environment (smoking) and defect size (deeper >5 mm and wider >3 mm defects being associated with more difficult root coverage). A number of specific surgical considerations were also identified as being important to success: flap thickness (≥ 1.1 mm), post-surgical position of the gingival margin (the higher the better) and maintaining a stable flap under low tension provided optimal wound healing. The importance of using biological agents (e.g. enamel matrix derivative or EMD) in conjunction with flap surgery was shown to be beneficial. The use of a modified coronally advanced tunnel flap approach in treating gingival recession was demonstrated, with the advantage of optimising tissue blending and aesthetics.

Unfortunately, objective information evaluating in which patients soft tissue augmentation should be performed before orthodontics is absent. However, the presence of dehiscence or fenestration is suspected; two potential options exist: avoid over-expansion of the arch, i.e. attempt to maintain the teeth within the dento-alveolar envelope by considering dental extractions or interproximal enamel reduction. In situations where we absolutely need to expand orthodontically out of the envelope, it would be wise to ask for soft tissue augmentation prior to treatment.

In terms of what is the best method(s) for recession coverage, there is a need to distinguish between single and multiple recessions. In respect of *single* gingival recession, a number of options exist for recession coverage, an EMD (Emdogain) with or without a connective tissue graft in conjunction with a coronally positioned flap [17, 18]. Good long-term (5 years) outcomes have been reported with connective tissue grafts and a coronally advanced flap [19]. Alternative methods include the envelope technique with connective tissue graft [20] or the laterally positioned flap with or without connective tissue graft. In the case of multiple recessions, the modified coronal advancement flap with or without graft is preferred in the maxilla [21], whilst in the mandible, its use in conjunction to a connective tissue graft should be considered [22]. In regard to the Miller's class III defect, the modified coronal advancement tunnel technique with connective tissue graft should be considered [22]. Whilst a free gingival graft can be used in both single and multiple gingival recessions, it is associated with high morbidity due to graft removal from the palate and sometimes necrosis of the graft. A frenectomy can also be considered. The importance of re-educating the patient in respect of their brushing technique was also highlighted, in conjunction to considering adjunctive cleaning aids, such as water picks and interdental toothbrushes.

## Conclusion

The consensus viewpoint from this day was that whilst controversy still exists in relation to the role of orthodontic treatment and gingival recession, there is a need to undertake a risk assessment and appropriate consent prior to the commencement of treatment. Awareness was required during treatment for any signs of gingival recession, and the most appropriate treatment mechanics designed to retain the roots within the alveolar bony envelope was the key. There remains a relatively weak evidence base for these decisions and further prospective; well designed trials are needed.

## Authors' information

MR is the president of the ASE membership group.
